# Update on the perioperative management of diabetes mellitus

**DOI:** 10.1016/j.bjae.2024.04.007

**Published:** 2024-05-31

**Authors:** J.A.W. Polderman, J. Hermanides, A.H. Hulst

**Affiliations:** Amsterdam University Medical Centres, Amsterdam, The Netherlands

**Keywords:** diabetes mellitus, glycaemic control, perioperative care

**Learning objectives**

By reading this article, you should be able to:

• Explain the mechanism of action of glucagon-like peptide-1 receptor agonists (GLP-1 RAs) and know the perioperative recommendation.
• Outline the indications and beneficial effects of sodium-glucose transporter 2 inhibitors (SGLT2Is) and the main risks of their use in the perioperative period.
• Understand the relevant characteristics of different types of diabetes.
• Discuss the limitations and concerns for clinicians and the patient on using a continuous glucose monitor (CGM) or continuous subcutaneous insulin infusion (CSII) pump in the perioperative period.

**Key points**

• Good preoperative assessment and planning are critical for the optimal perioperative management of diabetes mellitus.
• Clinicians must ascertain the type of diabetes in all patients, including children.
• Guidelines strongly recommend preoperative HbA1c measurements.
• No continuous glucose monitor or continuous subcutaneous insulin infusion pump has been certified for perioperative use.
• After surgery, a basal-bolus insulin regimen is preferable to a sliding-scale (short-acting, bolus-only) insulin protocol.

Diabetes mellitus is a public health concern, with a steadily increasing global prevalence. According to the International Diabetes Federation (IDF), ∼536 million adults (aged 20–79 yrs) worldwide were living with diabetes in 2019, representing a prevalence of 10.5%.^1^ Diabetes mellitus is more frequently prevalent in surgical patients compared with the general population, given the increased risk of surgical interventions in individuals with diabetes-related complications, although there are substantial differences between surgical specialties. A meta-analysis of 90 studies, including 866,427 surgical records, reported an overall prevalence of diabetes of 17%.^2^ The prevalence of diabetes was highest in patients presenting for cardiovascular surgery (up to 39%), followed by orthopaedic surgery.^2^ Among patients undergoing bariatric surgery, the prevalence of type 2 diabetes was 26%.^3^ The presence of diabetes in surgical patients is associated with an increased risk of perioperative complications, a prolonged hospital stay and higher rates of morbidity and mortality.^2^^,^^3^ Poor glycaemic control in diabetic surgical patients further exacerbates these risks.^2^^,^^3^ Studies have demonstrated a higher incidence of surgical site infections, delayed wound healing, cardiovascular events and respiratory complications in surgical patients with diabetes.^2^^,^^3^

## Pathophysiology

Glucose metabolism plays a vital role in energy production and maintenance of blood glucose concentrations within a narrow range. In healthy individuals, sodium-glucose transporter 1 (SGLT1) and glucose transporter (GLUT) enzymes facilitate glucose uptake in response to oral intake. Furthermore, glucagon-like-peptide-1 (GLP-1) is secreted by the intestinal L-cells in response to eating.^4^ Binding of GLP-1 to its receptors stimulates insulin secretion from the pancreas. Insulin allows glucose to be transported into the cells, where it undergoes a series of enzymatic reactions, collectively known as glycolysis, to produce energy such as adenosine triphosphate (ATP). Excess glucose is stored in the liver and skeletal muscle as glycogen (glycogenesis).

In people with diabetes mellitus, the regulation of glucose metabolism is impaired. The majority of patients presenting with impaired glucose control have type 2 diabetes mellitus (T2DM).^5^, ^6^, ^7^ In T2DM, the body develops insulin resistance, hampering glucose uptake into cells, or the pancreas fails to produce enough insulin to meet the body's demands. Nonetheless, around 10% of adult patients have T1DM or other less common forms of diabetes mellitus. In T1DM, the pancreas fails to produce insulin, which is caused by the (autoimmune) destruction of insulin-producing beta cells in the islets of Langerhans. Besides T1DM and T2DM, many other forms of DM with distinctive pathophysiology exist. Table 1 provides an overview of these different forms of diabetes, with defining characteristics and relevant points for perioperative management.

**Table 1:** Types of diabetes and specific concerns for the anaesthetist. CFRD, cystic fibrosis related diabetes; GDM, gestational diabetes mellitus; LADA, latent autoimmune diabetes in adults; MODY, maturity onset diabetes of the young; PTDM, post-transplant DM; T1DM/T2DM, type 1/2 diabetes mellitus.

Type of diabetes		Pathophysiology and clinical features	Insulin deficiency	Perioperative dysregulation	Perioperative concerns
1	T1DM	• Autoimmune destruction of pancreatic β-cells, leading to absolute insulin deficiency	Absolute	Common	• Hypoglycaemia is common • Consider referral to DM care physician • Always need exogenous insulin source (basal insulin, pump or i.v. drip)
2	T2DM	• Combination of insulin resistance and deficiency caused by diet, life-style and genetics	Relative	Depending on severity	• Associated comorbidities • Depending on severity
	LADA	• Autoimmune diabetes which does not manifest until adulthood. Clinically heterogenous group on the continuum between T1DM and T2DM	Variable	Variable	• Do not omit basal insulin, especially if anti-GADi titre is high • Few data regarding perioperative glucose control
3a	Monogenetic diabetes (e.g. MODY or neonatal diabetes)	• Rare forms of diabetes, typically as a result of genetic defects in β-cell function causing impaired insulin secretion. Clinical features depend on the subtype and genetic defect	Variable	Variable	• Clinically heterogenous • MODY subtype 2 (15–50%, Table 2) is generally mild. Manage other types as T1DM or T2DM depending on phenotype
3b	Pancreatic diabetes (e.g. pancreatitis) CFRD	• Pancreatitis leads to islet tissue fibrosis and destruction, resulting in insulin and glucagon deficiency.	Variable	Yes	• Marked glycaemic variability and possibly unpredictable response to exogenous insulin
3c	Endocrinopathy-related DM	• Insulin resistance and deficiency as a result of the excess release of counterregulatory hormones such as cortisol, GH/IGF-1 and catecholamines	Limited	Yes	• Commonly requires glycaemic monitoring and insulin (especially for phaeochromocytoma) • Beware of rebound hypoglycaemia after tumour resection
3d	Medication- related DM (e.g. glucocorticoid- induced) and PTDM	• Systemic corticosteroid treatment causes insulin resistance, increased gluconeogenesis and abnormal insulin secretion • PTDM is primarily caused by diabetogenic properties of the immunosuppressive agents	Limited	No	• Corticosteroid stress doses cause hyperglycaemia • Hyperglycaemia in PTDM is associated with risk of transplant rejection
4	GDM	• Diabetes first diagnosed during pregnancy. Associated with an increased risk of developing T2DM in later life	Limited	No	• Perioperative glycaemic target: 3.9–8.0 mmol L^−1^, as glucose ≥8.0 mmol L^−1^ may cause transient neonatal hyperinsulinism and neonatal hypoglycaemia

## Paediatrics

Diabetes mellitus is also one of the most common chronic diseases in children, and the incidences of both T1DM and T2DM in children are increasing. This is partly triggered by the increasing incidence of obesity in childhood. Currently, 20–40% of the patients with newly diagnosed T1DM are obese.^8^ The relation between obesity and T2DM is well established. Historically, T2DM accounted for 10% of paediatric patients; today, T2DM already accounts for >30% of paediatric patients in the USA.^8^ Although the number of patients with T1DM is increasing, the prevalence of children with T2DM has increased even more in the last two decades.^8^ Furthermore, obesity in children with impaired insulin secretion will lead to an earlier clinical manifestation of T1DM. Insulin needs are unmet because of obesity-induced insulin resistance, and obesity influences the progression of islet autoimmunity. This means that a child with DM will not necessarily have T1DM, and a child with obesity and DM will not necessarily have T2DM. Therefore, it is vital to pay close attention to the type of diabetes in children and adults, because requirements and management differ between types of diabetes (Table 1).

## Preoperative considerations and goals

One of the most important parts of perioperative care for patients with DM is a thorough preoperative assessment, including the adjustments to medication and a plan for in-hospital glucose control. A perioperative pathway for people with diabetes has the potential to increase efficiency and reduce waiting lists for elective surgery.^9^ Before surgery, we should focus on the assessment of the type of DM, antihyperglycaemic treatment, quality of glycaemic control and the severity of the diabetes-related complications such as cardiovascular disease, chronic renal failure, autonomic dysfunction and delayed gastric emptying. Secondary goals include optimising glycaemic control and general prehabilitation because of the increased risk profile of patients with DM. Patients have a higher prevalence of (advanced) coronary artery disease, increasing their risk of postoperative myocardial (silent) ischaemia and perioperative mortality after non-cardiac surgery (NCS).^10^ In addition, DM-induced autonomic dysfunction and peripheral neuropathy increase the risk of silent ischaemia.^11^ Furthermore, diabetes is a risk factor for stroke, congestive heart failure and surgical site infections. People with diabetes are considered good candidates for prehabilitation programmes because the commonly included diet and exercise interventions can improve glycaemic control and general health.^12^

### Haemoglobin A1c

In 2022, the European Society of Cardiology (ESC) published an update of their guideline for the assessment and management of patients undergoing NCS.^10^ This guideline includes two recommendations regarding diabetes and haemoglobin A1c (HbA1c).

(i) ‘In patients at high surgical risk, clinicians should consider screening for increased HbA1c before major surgery and improving preoperative glucose control.’ (recommendation class: IIa)^10^

However, screening for unknown diabetes mellitus through HbA1c measurements is not further substantiated, while the probability of diagnosing unknown diabetes is likely to depend on many factors. The incidence of undiagnosed diabetes differs significantly between regions or countries and is associated with income, resources, national guidelines and the quality of the (primary) healthcare system. This influences the cost-effectiveness of screening and should be implemented after such factors have been considered.

(ii) ‘In patients with diabetes or disturbed glucose metabolism, a preoperative HbA1c test is recommended if this measurement has not been performed in the previous three months. In case of HbA1c ≥8.5% (≥69 mmol mol^−1^), elective NCS should be postponed if safe and practical.’ (recommendation class: I)^10^

**Fig 1:**
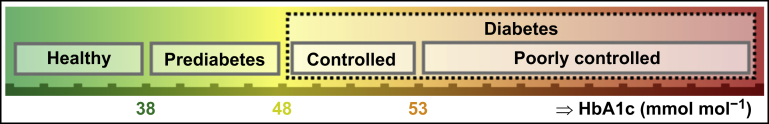
Interpretation of HbA1c values.

The available evidence for this strong recommendation by the ESC guideline is also poor. This is probably because the differentiation between association and causation has often been unclear. Factors extensively documented as independently associated with postoperative complications and worse outcomes include diabetes mellitus, increased preoperative HbA1c and perioperative hyperglycaemia. However, optimising preoperative glucose control in patients with a high HbA1c has not been studied in randomised controlled trials, and it is debatable whether this would prove an effective intervention. Nonetheless, HbA1c measurements provide valuable information on long-term glycaemic control in the previous months. Figure 1 provides an interpretation of HbA1c values. We support improving HbA1c concentrations in every patient, but are wary of the possible consequences before surgery. The COVID-19 pandemic demonstrated that postponement of surgery can seriously affect patients' health and quality of life.^13^ Therefore, the lack of evidence on preoperative HbA1c lowering should be weighed against the negative impact on patient satisfaction, health and quality of care when considering postponing surgery based on HbA1c concentrations.

### Non-insulin glucose-lowering medication

In addition to established medications such as metformin and sulfonylurea derivates, two newer non-insulin glucose-lowering drugs have been introduced and are gaining popularity. The mechanism of action of both GLP-1 receptor agonists (GLP-1 RAs) and SGLT2 inhibitors (SGLT2Is) are discussed below. A summary of the relevant US/UK guidelines on perioperative management of the most common non-insulin glucose-lowering medications is provided in Table 2.^14^^,^^15^

**Table 2:** Guidelines on perioperative management of the most commonly used non-insulin glucose-lowering medications. ∗If contrast medium is to be used and eGFR <60 ml min^−1^ 1.73 m^−2^, metformin should be omitted on the day of the procedure and for the following 48 h. ADA, American Diabetes Association Guideline Jan 2024; CPOC, Centre for perioperative Care, Academy of Medical Royal Colleges, Dec 2022.

Medication class (example)	Mechanism of action	Perioperative concerns	Perioperative management
**Biguanides (metformin)**	Decreases hepatic glucose production and increases muscle glucose absorption	(Lactic acidosis)	ADA: omit on day of surgery until oral intake resumed CPOC: continue∗
**Sulfonylureas (tolbutamide, glibenclamide, glimepiride)**	Stimulates β cell insulin secretion	Hypoglycaemia	ADA and CPOC: withhold on the day of surgery until oral intake resumed
**Thiazolidinediones (glitazones)**	Decreases insulin resistance	Fluid retention; hypoglycaemia	ADA: withhold on the day of surgery until oral intake resumed CPOC: continue
**Glucagon-like peptide-1 receptor agonists (GLP-1 RAs)** **(‘-natides’, ‘-glutides’)**	Stimulates insulin secretion and inhibits glucagon secretion, glucose-dependent	Delayed gastric emptying	ADA: withhold on the day of surgery until oral intake resumed CPOC: continue
**Dipeptidyl protein-4 inhibitors (DPP-4i)** **‘-gliptins’)**	Increases GLP-1 concentrations		ADA: withhold on the day of surgery until oral intake resumed CPOC: continue
**Sodium-glucose transport-2 inhibitors (SGLT2**Is**) (‘-gliflozins’)**	Induces renal glucose excretion	Euglycaemic ketoacidosis, diuresis, hypoglycaemia with insulin	ADA: withhold 72–96 h before surgery CPOC: withhold 48 h before surgery

#### Glucagon-like peptide-1 receptor agonists

Endogenous GLP-1 is a gut-derived incretin hormone that reduces glycaemia by stimulating insulin production and secretion from pancreatic β cells and by reducing glucagon secretion from α cells. In addition, GLP-1 inhibits gastric emptying and reduces appetite. Although this leads to less food intake, weight loss and improved glycaemic control, it is also responsible for the main adverse effect of nausea.

Notably, the pancreatic effects of GLP-1 are hyperglycaemia-dependent, making the risk for hypoglycaemia extremely low. Besides established efficacy in glycaemic control, enthusiasm for these medications increased with the findings of large cardiovascular outcome trials that found clear benefits of lower rates of myocardial infarction, stroke and revascularisation procedures. Initially, GLP-1 RAs came to the market as a second-line treatment option for T2DM, but currently, the indications are expanding to include weight loss in patients with obesity (regardless of T2DM). This field is rapidly developing with the introduction of dual and triple agonists (for a combination of GLP-1, GLP-2, glucagon and GIP [gastric inhibitory peptide]). The number of patients using a form of GLP-1 RAs is expected to increase significantly in the coming years, given the beneficial effects on diabetes-related complications and the expansion of the indication to weight control. Initially, withholding GLP-1 RAs was advised for in-hospital patients, whereas others are considering perioperative continuation, given the low risk of hypoglycaemia and improved glycaemic control,^14^^,^^16^^,^^17^ also because the advent of longer-acting (once-weekly) preparations makes adequate withholding more impractical.^18^^,^^19^ Drugs from this medication class all end with –natides or –glutides. The primary (adverse) effect of delayed gastric emptying raises concerns from anaesthetists because of the potential risk of aspiration and postoperative nausea and vomiting (PONV). Although GLP-1 RAs delay gastric emptying, this is most notable for the first postprandial hours and subsides as a result of tolerance and tachyphylaxis with ongoing treatment.^19^^,^^20^ Therefore, caution is needed about delayed gastric emptying in patients recently started on a GLP-1 RA, although after >12 weeks of treatment and after standard preoperative fasting times, gastric emptying is probably normal.^19^^,^^20^

#### Sodium-glucose transporter 2 inhibitors

Although SGLT2Is initially proved moderately effective in improving glycaemic control, subsequent large cardiovascular outcome trials in people with T2DM demonstrated that SGLT2Is improved major cardiorenal outcomes.^21^ Treatment with SGLT2Is reduces hospitalisation rates for heart failure and the progression of chronic kidney disease.^22^^,^^23^ Given the moderate improvements in HbA1c, the mechanisms of action on the heart and kidney are unlikely to be mediated by glucose control alone. Large randomised clinical trials in patients with heart failure (with preserved and reduced ejection fraction) and chronic kidney disease, all irrespective of a history of diabetes, demonstrated improved outcomes.^22^^,^^23^ Indications are expanding from T2DM to patients with heart failure and chronic kidney disease without DM.^22^^,^^23^ Although rare, the primary concern with SGLT2Is in the perioperative period is their association with euglycaemic ketoacidosis. This is an incompletely understood complication of using SGLT2Is, possibly precipitated by the surgical stress response. A retrospective review of 1307 patients on SGLT2Is undergoing surgical procedures found a euglycaemic diabetic ketoacidosis (DKA) incidence of 0.2% in non-emergent procedures and 1.1% for emergent procedures, given the inability to withhold SGLT2is in the latter group.^24^ Expert opinion and guidelines recommend withholding SGLT2Is perioperatively, although the recommended durations vary. The Centre for Perioperative Care (CPOC) recommends omission from the before surgery, whereas the ESC recommends 3 days before surgery.^10^^,^^15^ Drugs from this medication class all end with –gliflozins. Several studies are underway to assess starting an SGLT2I before surgery to prevent acute kidney injury (NCT05590143).

### Perioperative insulin treatment

Insulin is prescribed in different forms of preparations and dosing schemes. Available insulin preparations differ little pharmacodynamically but mostly in pharmacokinetics, ranging from (ultra-) short-acting to long-acting and mixed preparations, requiring between one to several injections daily or a continuous infusion via an insulin pump. The literature provides many recommendations on dose adjustments in the perioperative period. In clinical practice, we recommend adherence to one's institutional protocol for the perioperative treatment of diabetes, even though local protocols cause significant variability between hospitals, deviating from international guidelines.^25^ The literature does not support a specific protocol; for example, we refer to the US/UK anaesthesia societies supported guidelines.^14^^,^^15^^,^^26^ Fasting allows a preoperative reduction of insulin doses. The guidelines advise reducing long-acting insulin analogues to 75–80% of the usual doses and intermediate-acting by 50%.^14^^,^^15^^,^^26^ In patients with T1DM and T2DM with insulin therapy, especially when the fasting period exceeds one missed meal or when an insulin pump cannot be used, patients have to be switched from their insulin schedule to a form of i.v. insulin infusion. Also, in emergency surgery or when patients are persistently hyperglycaemic, it is recommended to switch to a form of i.v. insulin, such as a variable-rate i.v. insulin infusion (VRIII, e.g. in chapter 2 of the CPOC guideline^15^) or a combined glucose-insulin infusion. For safety, patients should always receive a glucose infusion alongside any VRIII. Iatrogenic hypoglycaemic events usually occur because the glucose substrate has been stopped, but the insulin infusion continued. To decrease this risk, one can add 1/8 of the total daily dose of insulin (minimum 6 units) to an infusion of glucose 5%, 500 ml at an infusion rate of 83 ml h^−1^. Historically, potassium was also added (GIK infusion). However, it has been shown that postoperative potassium concentrations are comparable between patients receiving a glucose-insulin infusion and a GIK infusion.^27^ To reduce medication and user error, each ward or hospital should have a protocol and adhere to it.^14^

On the day of surgery, safety concerns dictate that the emphasis should be on avoiding hypoglycaemia rather than hyperglycaemia. During anaesthesia and the recovery period the patient's capability to report the symptoms of hypoglycaemia is reduced, whereas hypoglycaemia, especially missed episodes, is more harmful than hyperglycaemia. The lower glycaemic target range is increased to 5.6 mmol L^−1^, thereby preventing more severe hypoglycaemia in fasting patients at risk because of reduced conscious states in the perioperative period.^14^ Hypoglycaemia in the perioperative period or intensive care (glucose <5.6 mmol L^−1^) is usually iatrogenic and associated with poor outcomes.^28^^,^^29^ Similar to the outpatient setting, it is best treated with i.v. glucose (e.g. 15 g), and then rechecked after 15 min (the 15–15 rule).^30^ Nonetheless, it is essential to never withhold insulin entirely in patients with T1DM or T2DM with high insulin dependency because this can lead to insulin deficiency and ketoacidosis.^5^, ^6^, ^7^

During surgery, glucose should be measured every 1–2 h, depending on the stability of the glucose value. Because of unreliable perfusion of subcutaneous tissue and the potential risk of insulin stacking when subcutaneous boluses are given more than once every 2–3 h, one should treat intraoperative hyperglycaemia (glucose >10 mmol L^−1^) with an i.v. correction bolus of insulin (sliding scale) or with adjustment of the VRIII. This is especially important in children with T1DM, and it is valuable to obtain the child's own correction factor to treat hyperglycaemia in the perioperative period. The correction factor or insulin sensitivity factor is the expected decrease in blood glucose after 1 unit of insulin. If the correction factor or insulin sensitivity factor is unavailable from the patient's records, it can be calculated by dividing 100 by the patient's average total daily dose of insulin. The result will give you the expected glucose decrease in mmol L^−1^ after 1 unit of insulin.

## Diabetes apparatus

### Continuous subcutaneous insulin pumps

The first insulin pump was developed by Dr Arnold Kadish in 1960. It took another 24 yrs before the first insulin pump became commercially available. Only after the publication of the DCCT trial in the 1990s (showing that intensive insulin treatment prevented diabetes-related complications) did continuous subcutaneous insulin infusion (CSII) device use start to increase in clinical practice.^31^ Since then, the percentage of patients using an insulin pump has increased steadily. Nowadays, more than half of the patients with T1DM use a CSII, but also the number of patients with T2DM using CSIIs is increasing. The T1DM exchange registry reported an increase in pump use from 57% to 64% from 2010 to 2018.^32^ The most significant increase in use was seen in children, of whom 60–70% are now treated with a CSII.^32^

A subcutaneous insulin pump attempts to mimic physiological insulin secretion with a continuous (ultra) short-acting insulin infusion and boluses at mealtimes. Both the basal rate and the boluses are easily adjustable, which allows more flexibility in insulin dosing. Using a pump significantly improves glycaemic control without an increase in hypoglycaemic events, while improving patients' satisfaction and quality of life.^33^

The manufacturers of continuous insulin pumps state that the device should not be used in the presence of imaging devices or electrocautery. However, switching a patient to an i.v. insulin infusion or multiple daily injections increases the risk of ketoacidosis and hypoglycaemia from potential miscalculations or delays in the initiation of an i.v. insulin infusion. Therefore, both adult and paediatric guidelines agree that an insulin pump may be used perioperatively unless there is an absolute contraindication (magnetic resonance imaging, anticipated significant fluid shifts). The rationale can be found in the guideline for perioperative care for people with diabetes mellitus undergoing elective and emergency surgery.^15^

#### Continuous glucose monitors

In parallel with the continuous subcutaneous insulin pump, more reliable subcutaneous continuous glucose monitors (CGMs) have become available to facilitate frequent glucose monitoring, mitigating the need for finger pricks. This led to a similar increase in the use of CGM, especially in children. Currently, >50% of patients under 12 yrs and around 30% of patients between 12 and 18 yrs with DM are using a CGM in daily life.^32^ All subcutaneous sensors have comparable accuracy and have been used successfully in many paediatric patients. However, during surgery and anaesthesia, physiological derangements such as hypotension and hypothermia potentially diminish perfusion of the subcutaneous tissue. In addition, pressure on the CGM device and the underlying tissue could influence readings. Finally, certain medications (e.g. paracetamol i.v.) can cause false high readings of the CGM with some types of sensor. Therefore, the accuracy of the CGM in the perioperative period is considered insufficiently reliable. However, the technology is evolving fast, and a small study with a flash glucose monitoring system showed promising results in critically ill patients.^34^ However, when leaving the CGM in place, we advise performing regular blood glucose checks to guide perioperative clinical decisions.

### Closed-loop systems

Manufacturers are trying to combine CGM, CSII and escape glucagon infusions in a closed-loop system, creating ‘the artificial pancreas’. The first hybrid closed-loop system has been commercially available since 2016. The pumps adapt the insulin infusion using the data from a CGM, the target range and mealtime information (the latter being entered by the patient). Perioperative, all the statements mentioned above apply to (hybrid) closed-loop systems. In research, closed-loop systems have been successfully used during surgery, in-hospital and non-critical care.^35^^,^^36^ In these studies, additional hourly point-of-care testing was used to monitor proper functioning.^36^ It is only possible and advisable when all parties involved desire and agree to continue the closed-loop system, know how it works and have a proper backup plan.

## Perioperative considerations related to anaesthesia

### Dexamethasone

Dexamethasone is used commonly as an antiemetic. As a potent glucocorticoid, it causes dose-dependent hyperglycaemia, most notable on the first day after surgery, with a peak effect between 8 and 16 h after dosing. However, the clinical significance was largely unknown until the Perioperative Administration of Dexamethasone and Infection (PADDI) trial was published.^37^ The investigators reported that in >8800 patients randomised to dexamethasone 8 mg *vs* placebo, there was no difference in the primary endpoint of surgical site infections (8.1% for dexamethasone *vs* 9.1% for placebo, 95% confidence interval (CI)=−2.1 to 0.3). A subgroup analysis of the cohort of 1065 patients with diabetes mellitus reveals a similar result (11.2% *vs* 13.6%, 95% CI=−6.6 to 1.6). Therefore, although dexamethasone increases perioperative hyperglycaemia, it does not increase the risk of surgical site infections. There are few data of similar quality on other postoperative complications, but surgical site infections are one of the most common, relevant postoperative complications linked to diabetes and perioperative hyperglycaemia. Meanwhile, the PADDI trial confirmed the effectiveness of dexamethasone for PONV (dexamethasone 42% *vs* placebo 54%, Risk Ratio (RR)=0.78, 95% CI=0.75–0.82).^37^ These results should also reassure clinicians using dexamethasone for PONV in patients with DM.

### Magnesium

A double-blinded RCT (*n*=122) in diabetic patients compared a continuous infusion of magnesium sulphate at 15 mg kg^−1^ h^−1^ from 20 min before surgery until 24 h after surgery with a control group receiving i.v. saline 0.9%.^38^ In the magnesium group, blood glucose and insulin requirements decreased compared with controls (mean postoperative blood glucose was 8.8 (1.5) *vs* 9.7 (1.5) mmol L^−1^).^38^ It is hypothesised that magnesium enhances blood glucose control through several mechanisms: increasing the affinity of insulin to its receptors, increasing pancreatic insulin secretion, potentiating insulin-mediated glucose uptake, regulating glycogenolysis and glucose output in the liver, the release of catabolic hormones and glucose translocation into the cell.^38^

### Regional anaesthesia

Diabetic neuropathy is common, caused by multiple contributing pathways, primarily triggered by persistent high blood glucose concentrations (chronic hyperglycaemia). This leads to inflammation and oxidative stress, causing changes in the small blood vessels, reduced blood supply to specific areas (local ischaemia) and decreased speed of nerve signal transmission (axonal conduction velocity). When considering regional anaesthesia in patients with diabetic neuropathy, one should be aware that the affected nerves are more sensitive to local anaesthetic agents, resulting in a longer duration of nerve block.^39^ There have been suggestions that local anaesthetics could be more toxic for affected nerves, but the evidence is inconclusive. Neuropathy should not therefore be a reason to deny regional anaesthesia to patients with a valid indication.^39^ One should be cautious of performing the block with electric stimulation only, as the threshold of the affected nerves for electric stimulation is significantly increased, which theoretically increases the risk of needle trauma.^39^ Finally, when peripheral nerve catheters are used, one should be aware of an increased risk of catheter-related infections.

## Future directions

### Preoperative carbohydrate loading

Preoperative carbohydrate loading (CHL) has been integrated into numerous guidelines for enhanced recovery after surgery (ERAS) protocols.^40^ The principal objective of CHL is to prevent the fasting state, thereby inhibiting catabolic pathways. Evidence suggests that CHL reduces cortisol, interleukin-6 and insulin resistance, attenuating the surgery-associated inflammatory response.^40^ Furthermore, CHL has been shown to enhance postoperative patient comfort by alleviating hunger, thirst, malaise, anxiety and nausea.^40^ There is uncertainty regarding its safety profile in patients with DM, as CHL may potentially increase the risk of aspiration resulting from delayed gastric emptying or gastroesophageal reflux. However, small studies have assessed gastric volume with ultrasound and found no difference between patients with T2DM receiving CHL and those without T2DM receiving CHL 2 h before surgery.^41^ Furthermore, small studies showed promising results comparable to improved glycaemic control after CHL in patients with T2DM when insulin was prescribed to cover the carbohydrate load.^41^

### HbA1c-adjusted glycaemic targets

For many years, we have tried to design ‘one-fits-all’ protocols for patients with diabetes. However, as we discussed earlier, the morbidly obese T2DM patient with a very low insulin sensitivity is not at all comparable to a T2DM patient only taking metformin or to the T1DM patient. Individualised care is ideal for many situations, and we should aim for this direction, in glycaemic control. As an example, we know that chronic essential hypertension shifts a patient's normal homeostasis of blood pressure control. In such patients, the thresholds for acute hypotension and hypertension are moved upwards. Therefore, perioperatively, we adjust our target mean arterial pressure (MAP) as a percentage of MAP at presentation or home. Similarly, chronic hyperglycaemia should affect our perioperative goals and shift the upper and lower bounds of our target range upwards. We do not have randomised clinical trials to show this practice improves outcomes, but there is indirect evidence to consider such an approach. As well documented, acute hyperglycaemia is related to worse outcomes, such as ICU mortality.^42^^,^^43^ However, this relationship dissolves in patients with pre-existing longer-term hyperglycaemia (HbA1c >53 mmol mol^−1^), suggesting that hyperglycaemia is likely to be only a risk factor for patients with glucose control that is usually well regulated.^43^ The inpatient specialised diabetes team could be essential in managing diabetes in the perioperative process.^9^ Future studies should address this personalised approach to glycaemic control.

## Postoperative management

Surgical site infections are the most commonly cited postoperative complications related to perioperative glucose control. Surgical stress leads to hyperglycaemia and impedes postoperative glucose control in patients with DM, which increases the risk of postoperative complications, especially surgical site infections. A meta-analysis showed that postoperative treatment with a variable rate insulin infusion with intensive monitoring in the ICU resulted in lower mean glucose values and reduced surgical site infections. Furthermore, small intervention studies on regular postoperative wards found that a sliding scale regimen led to increased mean glucose values and more surgical site infections when compared with a basal-bolus regimen.^44^ As such, the American Diabetes Association (ADA) guideline recommends a basal-bolus regimen in the postoperative ward in favour of sliding-scale insulin only.^14^ For clarity, sliding-scale insulin refers to a protocol prescribing single bolus, correctional doses of short-acting insulin without changes to continuous or long-acting insulin doses. Many hospitals still use sliding-scale insulin regimens despite their ineffectiveness in achieving adequate in-hospital glucose control, possibly out of fear of hypoglycaemia. Reassuringly, the risk of severe hypoglycaemia (<2.3 mmol L^−1^) is comparable between sliding scale and basal-bolus regimens. Once patients have adequate oral intake and are ready for discharge, they can return to their regular medication.

## Diabetic emergencies

Diabetic ketoacidosis and hyperglycaemic hyperosmolar syndrome (HHS) are two serious complications of diabetes mellitus. Diabetic ketoacidosis primarily occurs in patients with T1DM but can also affect those with T2DM. It develops because of insulin deficiency, leading to hyperglycaemia, increased lipolysis and ketone production. The lack of insulin causes a shift in metabolism, with the body relying on fat breakdown for energy. Ketones accumulate in the blood, leading to metabolic acidosis. Approximately one-third of children diagnosed with T1DM present with DKA at the time of diagnosis.^45^ The incidence tends to be higher in younger children, with the peak occurring between the ages of five and seven.^45^ Symptoms include the classical signs of diabetes (polyuria, polydipsia and weight loss) but can mimic an upper respiratory chest infection (tachypnoea) or gastroenteritis (abdominal pain, nausea, vomiting). A high degree of suspicion is warranted in toddlers, teenagers (with a known history of diabetes) and patients with an insulin pump. Keep in mind that during fluid resuscitation (paediatric) patients are at risk for cerebral oedema.

Hyperglycaemic hyperosmolar syndrome is typically observed in patients with T2DM, especially older adults. It arises from severe hyperglycaemia and extreme dehydration. Unlike in DKA, insulin still circulates in HHS, preventing ketone production. However, insulin is insufficient for adequate glycaemic control, leading to more pronounced hyperglycaemia (ADA diagnostic criteria for DKA include a glucose >13.9 mmol L^−1^ compared with >33.3 mmol L^−1^ for HHS).^45^ Hyperglycaemic hyperosmolar syndrome is further characterised by hyperosmolality (resulting from increased solute concentration), a relative lack of ketones and acidosis (pH>7.3) and mental changes. Hyperglycaemic hyperosmolar syndrome is more common in adults, particularly in elderly individuals with comorbidities such as hypertension and kidney disease. Treatment of DKA and HHS prioritises the correction of dehydration with saline 0.9%, followed by moderate glycaemic control (initially targeting blood glucose 10–15 mmol L^−1^) and supplementation of potassium with frequent monitoring of osmolality, glycaemia and potassium.^45^

## Conclusions

Diabetes is one of the most common comorbidities in patients presenting for surgery. Besides T2DM, other forms, such as T1DM, have specific clinical features and perioperative concerns. Thorough *preoperative* evaluation of a patient's antihyperglycaemic treatment and long-term regulation (HbA1c), together with possible diabetes-related complications, is recommended. Clinicians should adhere to local guidelines for perioperative antihyperglycaemic treatment and insulin schedules. Local guidelines should be updated frequently, as the evidence is evolving quickly. Soon, we expect updates on the perioperative use of GLP1-agonists and SGLT-2 inhibitors. Shared decision-making is advised when there is a wish to continue using subcutaneous insulin pumps or continuous glucose monitoring devices *intraoperatively.* Finally, a basal-bolus regimen in the ward leads to better glycaemic control compared with a sliding scale regimen in the postoperative period.

## Declaration of interests

The authors declare that they have no conflicts of interest.

## Funding

This project has received funding from the European Union's Horizon 2020 research and innovation programme under the Marie Skłodowska-Curie grant agreement number 101024833.

## MCQs

The associated MCQs (to support CME/CPD activity) will be accessible at www.bjaed.org/cme/home by subscribers to *BJA Education*.

## Acknowledgements

This publication is part of the project NEPHRITIC (project number 452020104) of the research programme Rubicon, financed by the Dutch Research Council (NWO).
