# Erratum to ‘Update on the perioperative management of diabetes mellitus’ [BJA Education 24 (2024) 261–269]

**DOI:** 10.1016/j.bjae.2026.07.004

**Published:** 2026-09-01

**Authors:** J.A.W. Polderman, J. Hermanides, A.H. Hulst

**Affiliations:** Amsterdam University Medical Centres, Amsterdam, The Netherlands

The publisher regrets that a word was omitted in the tenth sentence of the section ‘Sodium-glucose transporter 2 inhibitors’. The correct sentence should read:

The Centre for Perioperative Care (CPOC) recommends omission from the day before surgery, while the European Society of Cardiology (ESC) recommends three days preoperatively.

The publisher would like to apologise for any inconvenience caused.

